# Retronasal Olfactory Function After Olfactory Training: A Prospective Single‐Arm Study

**DOI:** 10.1111/coa.70156

**Published:** 2026-08-12

**Authors:** Michal Pieniak, Maher Abou Nasr, Selin Bayindir, Thomas Hummel

**Affiliations:** ^1^ Smell & Taste Clinic, Department of Otorhinolaryngology Technical University of Dresden Dresden Germany; ^2^ Institute of Psychology University of Wroclaw Wroclaw Poland; ^3^ Department of Otorhinolaryngology Knappschaftskrankenhaus Gelsenkirchen Germany

**Keywords:** aroma, olfactory dysfunction, orthonasal olfaction, retronasal olfaction, smell training

## Abstract

**Background:**

Retronasal olfaction is essential for aroma perception. Its impairment in individuals with olfactory dysfunction (OD) negatively affects dietary behaviour and quality of life. Regular exposure to odours in a form of olfactory training (OT) is used to rehabilitate the sense of smell. However, retronasal recovery following OT remains insufficiently characterised.

**Objective:**

To verify whether OT improves retronasal odour identification and whether such improvement is related to orthonasal odour identification recovery.

**Study Design:**

Prospective, single‐arm clinical study.

**Methods:**

Fifty‐three patients with OD (predominantly post‐COVID‐19) completed at least 3 months of OT (conducted bi‐daily with four odours). Retronasal function was assessed with the ThreeT test and orthonasal function with the Sniffin’ Sticks battery before and after training.

**Results:**

OT significantly improved both retronasal (9.40 ± 0.42 vs. 10.85 ± 0.48; *p* < 0.001) and orthonasal odour identification (8.75 ± 0.50 vs. 10.53 ± 0.50; *p* < 0.001). Changes in retronasal performance positively, moderately correlated with changes in orthonasal identification (*r* = 0.38, *p* = 0.006). Clinically meaningful retronasal improvement was associated with a higher likelihood of orthonasal recovery, *χ* ^2^(1) = 5.08, *p* = 0.024; OR = 4.00 (95% CIs: 1.16–13.83).

**Conclusions:**

OT improves retronasal odour identification. The mechanisms of orthonasal and retronasal recovery may be partially overlapping.

## Key Points

- Olfactory training improves retronasal odour identification in patients with olfactory dysfunction.
- Improvement in retronasal odour identification parallels improvement in orthonasal odour identification.
- Clinically meaningful retronasal improvement is linked to increased likelihood of orthonasal recovery.
- Mechanism responsible for retronasal and orthonasal improvements is likely to partially overlap.
- Findings should be interpreted cautiously due to the absence of a non‐training control group.

## 1 Introduction

Olfactory dysfunction (OD) affects millions of people worldwide and may result from viral infections like COVID‐19, chronic rhinosinusitis, head trauma and neurodegenerative conditions [1]. Patients with OD often experience alterations in the perception of flavour and modifications of eating habits, insecurity in social interactions and accidents caused by the inability to detect odours signalling threats, such as smoke or leaking gas [2, 3]. All these factors contribute to development of depressive symptoms and decreased quality of life [4].

Retronasal olfactory perception is evoked by odourous molecules reaching olfactory epithelium via the mouth, oropharynx and nasopharynx and is strongly linked to aroma experience and sensory evaluation of food [5]. In people with OD, impairment of retronasal odour identification is more strongly related to one's life quality than impairment of orthonasal olfactory functions [6]. Despite its relevance, retronasal olfaction has been studied less extensively than orthonasal olfaction, partially due to the limited availability of retronasal olfactory tests [7].

Olfactory training (OT) is a recommended method of smell rehabilitation in patients with OD [1]. Studies show that OT may trigger the regenerative capacity of olfactory epithelium and lead to functional and structural changes in the central olfactory system, eventually influencing olfactory perception [8]. OT positively affects orthonasal olfactory function, with more pronounced effects observed in odour discrimination and odour identification tests, and smaller effects in olfactory threshold [9]. However, the effects of OT on retronasal olfactory function are much less studied. One study demonstrated beneficial effects of OT on retronasal odour identification ability in people with COVID‐19‐related OD [10], however, patients with other aetiologies of OD did not improve in retronasal odour identification [11].

It remains poorly understood how recovery of retronasal odour identification, which is essential for aroma perception, is related to improvement of orthonasal odour identification. This study investigated this in patients with different aetiologies of OD. We employed a recently developed test for aroma perception—the retronasal odour identification ThreeT test [12]—to assess changes in retronasal olfaction following OT and verify how they relate to orthonasal improvement. We hypothesised that OT would increase retronasal odour identification ability.

## 2 Materials and Methods

### 2.1 Study Design and Ethical Statement

The study was a prospective, single‐arm clinical study conducted to investigate changes in retronasal olfactory function in patients with OD. The data collection started in April 2024 and ended in April 2025. The study has been conducted according to the Declaration of Helsinki on Biomedical Studies Involving Human Subjects guidelines. All participants provided informed, written consent. The study was approved by the ethics committee at the Dresden University of Technology (decision: BO‐EK‐3012024). All information is reported according to the STROBE guidelines [13].

### 2.2 Participants

Participants were recruited from the patient pool at Smell and Taste Clinic, Department of Otorhinolaryngology at the Dresden University of Technology, as well as through flyers. Inclusion criteria were: presenting with subjective OD, being mentally able to consent, and being 18 years or older. Exclusion criteria were pregnancy, smoking, chronic rhinosinusitis and chronic diseases such as chronic kidney disease. No a priori sample size calculation was conducted. Instead, all eligible participants willing to participate in the study within the recruitment period were enrolled (*n* = 65). Of these, 12 did not complete the follow‐up assessment.

### 2.3 Procedure

The study began with an initial consultation at the outpatient smell and taste clinic, where potential participants were informed about the study. During this initial visit, the participants filled in a medical history comprising demographic information, past medical history and questions for olfactory or gustatory dysfunction. Individuals who met the study's inclusion criteria and provided written informed consent completed the ThreeT retronasal odour identification test [12], and the orthonasal Sniffin’ Stick test [14]. Participants diagnosed with OD were advised to complete OT, after which a follow‐up assessment was conducted. The second session involved repeated olfactory tests to evaluate progress.

### 2.4 Methods

#### 2.4.1 Retronasal Olfactory Function

The ThreeT retronasal odour identification test uses 20 flavoured tablets (e.g., apple, lemon, banana and cinnamon), with 500 mg of sorbitol used as an inert carrier (Sentosphere, Paris, France) [12, 15]. During the examination, the participant is asked to block their nose, chew a tablet for 15–20 s to release the aroma. Then, the participant exhales through the nose to activate retronasal olfaction. Afterwards, the participant is asked to identify the correct odour from a list of four items. After each trial, the mouth is rinsed to ensure that residual aroma does not influence upcoming trials. Each correct answer is counted as one point, with a total possible score of 20.

#### 2.4.2 Orthonasal Olfactory Function

The Sniffin’ Sticks test comprises tests for olfactory threshold (T), discrimination (D) and identification (I) [14]. Olfactory threshold measures the lowest concentration of phenyl ethyl alcohol the participant can detect (starting from the 4% dilution and further diluted in 1:2 steps in propylene glycol, 16 steps in total). Odour discrimination test measures the ability to distinguish between different suprathreshold odours. Odour identification test measures the ability to recognise a suprathreshold odour from a set of four descriptors. A total score in the threshold test ranges from 1 to 16, and in the discrimination and identification tests from 0 to 16, with higher scores indicating better olfactory functions. Scores from the three subtests add up to the overall TDI score ranging from 1 to 48. Based on the normative data, patients with TDI score < 30.75 points were classified as hyposmic, and with scores of 16 or less points as anosmic [16]. Detailed description of the Sniffin’ Sticks administration protocol has been described by Hummel et al. [14].

#### 2.4.3 OT

OT is based on regular exposure to odorants representing different categories of odours. Rose (floral), eucalyptus (resinous), lemon (fruity) and clove (spicy) were used as training odours [17]. Participants were asked to mindfully smell these odorants for 30 s each, twice daily, for at least 3 months until the follow‐up visit.

### 2.5 Statistical Approach

Data were analysed jamovi (version 2.6.45.0) and visualised with R. To assess changes in odour identification, we used a repeated‐measures ANOVA (rm‐ANOVA) with two within‐subject factors: time (baseline, follow‐up) and odour identification test (retronasal ThreeT, orthonasal Sniffin’ Sticks identification). Type of odour identification test was used as a within‐subject factor as it would allow us to directly test a Time‐by‐Test interaction potentially indicating different recovery trajectories depending on the odour administration route. Partial eta squared (*η* ^2^_p_) was calculated as effect size. The effect size is reported without confidence intervals (CIs) as their estimation in repeated‐measures designs is not standardised for *η* ^2^_p_. Pearson's correlation was used verify relationship between baseline–follow‐up differences in the retronasal ThreeT and orthonasal Sniffin’ Sticks identification tests, as well as to test the association between baseline score and the subsequent change within ortho‐ and retronasal domains. Chi‐square (*χ* ^2^) test and odds ratio (OR) were used to verify whether obtaining a minimal clinically important difference (MCID) in the retronasal ThreeT test was related to increased frequency of obtaining MCID in the orthonasal Sniffin’ Sticks identification tests. For both tests, change of at least three points was treated as MCID [18]. Improvement in all orthonasal functions (the TDI score) was tested with a paired sample *t*‐test. In all analyses the significance level was set to *α* = 0.05 (two‐tailed). Descriptive statistics are presented as means (*M*) and standard deviations (SD); estimated marginal means for the rm‐ANOVA model are presented alongside standard errors (SE).

Whilst no a priori sample size calculation was conducted, we ran sensitivity analysis after the study completion using G*Power [19]. For a rm‐ANOVA with one group, with *α* = 0.05, the final sample size of 53 participants, and correlations between repeated measures ranging from *r* = 0.67 to *r* = 0.82, with power of 0.80 we could reliably detect small‐to‐moderate main effects between *f* = 0.16 and *f* = 0.12 (*η* ^2^_p_: 0.01–0.03), and small‐to‐moderate interaction effects between *f* = 0.13 and *f* = 0.10 (*η* ^2^_p_: 0.01–0.02).

## 3 Results

A total of 53 participants aged 25–86 years (*M* = 59.32, SD = 12.46; 25 women) completed OT and attended the follow‐up assessment. Regarding the aetiology, 47 participants had post‐COVID‐19 OD, 2 had post‐traumatic OD and 4 had idiopathic OD. Of these, 16 (30.2%) had TDI score in an anosmic range, 35 (66%) in a hyposmic range and 2 (3.8%) in a normosmic range. Descriptive statistics of the psychophysical olfactory tests are presented in Table 1.

**TABLE 1:** Descriptive statistics of the sample who completed olfactory training (*n* = 53).

	Mean	Median	SD	Minimum	Maximum
Age	59.32	61	12.46	25	86
Olfactory dysfunction duration (months)	31.47	36	21.40	3	108
Olfactory training duration (months)	4.36	4	0.71	2	7
Threshold—Baseline	3.44	2.50	2.70	1	9.50
Discrimination—Baseline	8.92	9	2.72	4	15
Identification—Baseline	8.75	9	3.64	0	16
TDI—Baseline	21.13	20.50	7.25	8	34.50
ThreeT—Baseline	9.40	10	3.06	3	15
Threshold—Follow‐up	3.57	1.75	3.16	1	11.50
Discrimination—Follow‐up	11.19	12	3.78	3	16
Identification—Follow‐up	10.53	11	3.67	2	16
TDI—Follow‐up	25.34	26.50	8.66	8	41.50
ThreeT—Follow‐up	10.85	11	3.53	2	18

The rm‐ANOVA model showed a significant effect of time, *F*(1, 52) = 32.55, *p* < 0.001, *η* ^2^_p_ = 0.39, but not a significant effect of odour identification test, *F*(1, 52) = 2.29, *p* = 0.137, *η* ^2^_p_ = 0.04, or an interaction effect between time and test *F*(1, 52) = 0.70, *p* = 0.408, *η* ^2^_p_ = 0.01. Post hoc tests showed that scores in the retronasal ThreeT test significantly increased over time (Baseline: *M* = 9.40, SE = 0.42; Follow‐up: *M* = 10.85, SE = 0.48; *p* < 0.001) and so did scores in the orthonasal Sniffin’ Sticks identification test (Baseline: *M* = 8.75, SE = 0.50; Follow‐up: *M* = 10.53, SE = 0.50; *p* < 0.001) (Figure 1A,B).

**FIGURE 1:**
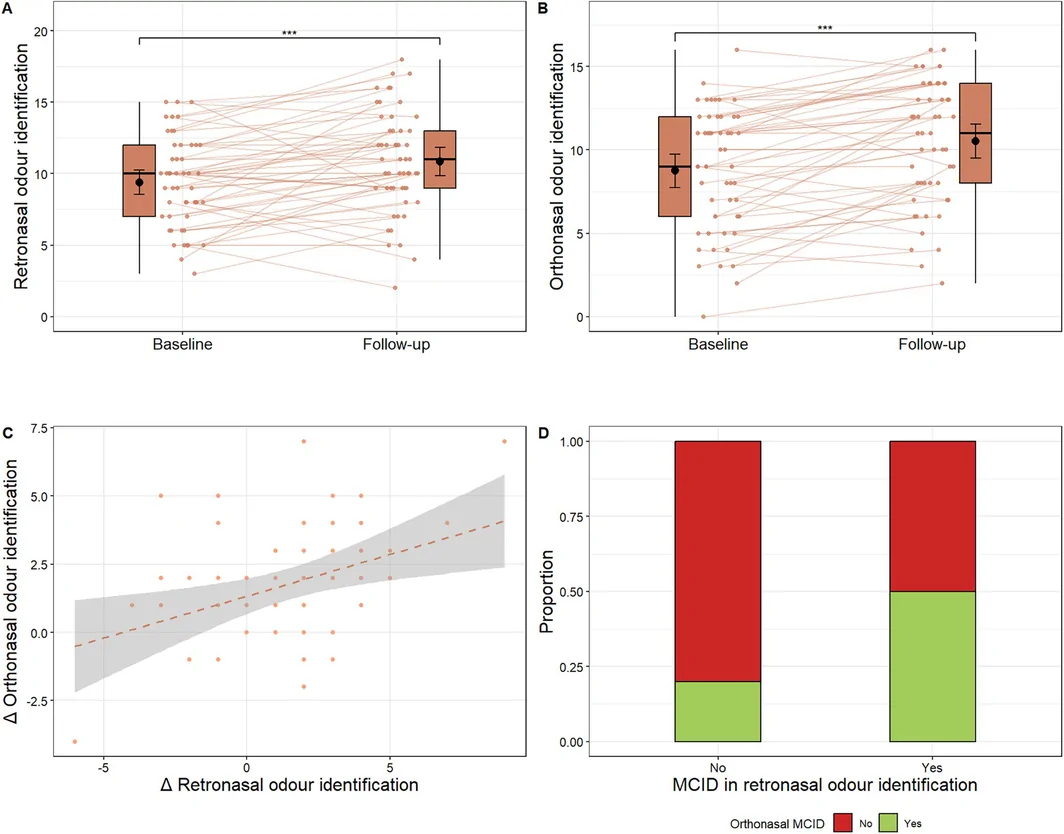
Scores in the retronasal ThreeT (A) and orthonasal Sniffin’ Sticks identification (B) tests increased between baseline and follow‐up. Dots inside the boxplots represent means and 95% confidence intervals. Baseline–follow‐up differences in the scores of both tests were positively correlated (C). The regression line is presented with standard error band. Participants who obtained a minimal clinically important difference (MCID) in the ThreeT test were more often exhibiting MCID in the Sniffin’ Sticks test (D). ***p < 0.001.

Changes in the retronasal ThreeT test scores positively and moderately correlated with changes in the orthonasal Sniffin’ Sticks identification test scores, *r* = 0.38, *p* = 0.006 (Figure 1C). There was a significant association between MCID in the retronasal and orthonasal identification tests, *χ* ^2^(1) = 5.08, *p* = 0.024, OR = 4.00 (95% CIs: 1.16–13.83). Specifically, amongst participants who obtained MCID in the retronasal ThreeT test (*n* = 18), 50% obtained MCID also in the orthonasal Sniffin’ Sticks identification test and 50% did not improve in the orthonasal Sniffin’ Sticks identification test. However, in the group who did not obtain MCID in the retronasal ThreeT test (*n* = 35) only 20% obtained MCID also in the orthonasal Sniffin’ Sticks identification test, whereas 80% did not (Figure 1D). Baseline orthonasal odour identification score was weakly, positively correlated with change in this score over time (*r* = −0.29, *p* = 0.034), indicating that patients with lower baseline scores showed greater improvement over time. A similar trend was observed for the retronasal odour identification score; however, it did not reach the significance level (*r* = −0.26, *p* = 0.059).

Participants improved in the overall orthonasal olfactory functions (TDI score), *t*(52) = 5.24, *p* < 0.001, Cohen's *d* = 0.72 (Baseline: *M* = 21.13, SD = 7.25; Follow‐up: *M* = 25.34, SD = 8.66). Following OT, based on the TDI score 11 patients (20.8%) were classified as anosmic, 25 (47.2%) as hyposmic and 17 (32.1%) as normosmic.

## 4 Discussion

This study investigated whether OT influences retronasal odour identification and whether changes in the retronasal olfaction are related to changes in the orthonasal olfaction. We found that retronasal odour identification ability increased in patients with OD following OT, replicating previous findings in post‐COVID‐19 OD [10]. Therefore, we contribute new evidence showing the potential of OT to recover aroma perception in individuals with olfactory impairment.

Additionally, we found that olfactory retronasal improvement is related to orthonasal olfactory improvement. This effect manifested in two ways—as a positive correlation between changes in retronasal and orthonasal odour identification scores and as increased frequency of clinical improvement in orthonasal odour identification in individuals who showed improvement in retronasal olfaction. This suggests that the mechanisms involved in odour identification recovery overlap between the retronasal and orthonasal domains. Considering the correlation was moderate and that the retro‐ and orthonasal systems partially rely on different anatomical structures and neural pathways [20, 21], there might be additional domain‐specific mechanisms of recovery that should be further investigated in follow‐up neuroimaging and molecular studies.

Baseline orthonasal odour identification scores were negatively correlated with the change in the identification score over time, showing that people with low baseline scores showed greater improvement. This effect has been characterised in the literature before [22]. Here, we demonstrate that this effect is present with comparable strength (although statistically on a trend level) in retronasal odour identification recovery, showing one more similarity in the recovery trajectories of ortho‐ and retronasal odour identification abilities.

Individuals with olfactory impairment often show altered dietary behaviours and decreased importance of food sensory perception, partially arising from disturbed aroma perception [23, 24]. A recent OT study in a non‐clinical ageing population showed no effects of OT on dietary behaviours [25], however, the effects might be related to preserved retronasal functions in healthy aging individuals [26]. How OT‐induced changes in retronasal odour identification in individuals with OD translate to dietary behaviours is yet to be verified.

The study is limited by the small sample size (not allowing us to detect smaller, more subtle effects, such as the correlation between baseline ThreeT scores and change in these scores over time) and the absence of a control group which was not involved in OT (allowing comparison to spontaneous recovery). Future studies using a larger cohort are needed to replicate our findings and evaluate the stability of retronasal olfactory improvement.

## 5 Conclusion

OT improved retronasal odour identification ability in individuals with OD. This improvement was positively related to improvement of orthonasal odour identification ability, suggesting at least partial overlap of the recovery mechanisms between retro‐ and orthonasal domains.

## Author Contributions

**Michal Pieniak:** data analysis, conceptualisation, methodology, visualisation, writing – original draft. **Maher Abou Nasr:** data analysis, writing – original draft. **Selin Bayindir:** investigation, writing – review and editing. **Thomas Hummel:** conceptualisation, project administration, methodology, resources, supervision, writing – review and editing.

## Funding

The study received in‐house funding by the Department of Otorhinolaryngology, TU Dresden. M.P. was supported by the National Science Centre Grant (OPUS #2020/37/B/HS6/00288).

## Ethics Statement

The study was approved by the Ethics Committee at the Dresden University of Technology (decision: BO‐EK‐3012024).

## Consent

All participants provided an informed, written consent.

## Conflicts of Interest

The authors declare no conflicts of interest.

## Data Availability

The data that support the findings of this study are available from the corresponding author upon reasonable request.
